# A rapid screening tool for fatigue impact in multiple sclerosis

**DOI:** 10.1186/1471-2377-6-27

**Published:** 2006-08-17

**Authors:** Daphne Kos, Guy Nagels, Marie B D'Hooghe, Marijke Duportail, Eric Kerckhofs

**Affiliations:** 1 Department of Rehabilitation Research, Vrije Universiteit Brussel, Laarbeeklaan 103, 1090 Brussels, Belgium; 2 Department of Neurology, National MS Centre Melsbroek, Belgium; 3 Department of Occupational Therapy, National MS Centre Melsbroek, Belgium

## Abstract

**Background:**

Fatigue is a common complaint in multiple sclerosis (MS) and often interferes with daily functioning. Both clinicians and researchers may need to detect high levels of fatigue impact using a time and effort efficient tool. This study evaluates the psychometric properties of a rapid screening instrument for fatigue impact in multiple sclerosis.

**Methods:**

Three visual analogue scales (VAS) for assessing the impact of fatigue were developed. Sixty two subjects with definite MS (mean age 52 +/- 10.5 years; 29 women) and 24 healthy controls (mean age 52 +/- 14 years; 13 women) completed all VAS scales (range 0–100), the Fatigue Severity Scale (FSS) (range 7–63), the Modified Fatigue Impact Scale (MFIS) (range 0–84) and the Guy's Neurological Disability Scale (GNDS) (range 0–5). All tests were repeated with an interval of maximum three days.

To evaluate the reproducibility, intraclass correlations (ICC) were calculated, based on one-way analysis of variance for repeated measurements. Validity was considered by means of correlation coefficients. ROC analysis was used to determine the accuracy of the VAS scales.

**Results:**

The ICC of the VAS scales ranged from 0.68 to 0.69. VAS scales showed low to moderate correlation with FSS, MFIS and GNDS (Kendall's tau 0.23–0.45) and were not related with physical or cognitive performance, or with depression. All VAS scales were able to discriminate between subjects with MS and controls. Twenty five subjects with MS had a Fatigue Severity Scale score of 36 or more and were classified into the "fatigue" group. ROC analysis showed that VAS_1 is most useful to classify subjects in the "fatigue" group. A cut-off value of VAS_1 of 59 displayed 76% sensitivity and 72% specificity. When using the MFIS score of 40 or more to classify the groups, VAS_1 remained the strongest tool, with 81% sensitivity and 77% specificity at a cut-off value of 59.

**Conclusion:**

The VAS for the impact of fatigue on daily life (VAS_1) is a moderately reliable, though valid and useful tool to screen rapidly for fatigue impact in multiple sclerosis. A cut-off value of 59 satisfactorily classifies individuals having severe fatigue with a high impact on daily life. In clinical practice, a more comprehensive assessment of fatigue and the impact on daily life is recommended.

## Background

In multiple sclerosis, fatigue limits the daily life functioning to a great extent [1]. For example, fatigue is one of the major reasons for unemployment in multiple sclerosis [2].

The influence of fatigue on performance is commonly quantified by self-report instruments. Several instruments have been developed and evaluated, though no "golden standard" exists. The choice of an instrument mainly depends on the purpose, e.g. Fatigue Severity Scale (FSS) is used to assess the severity of fatigue [3]. Having severe fatigue does not necessarily mean a high degree of limitation in his or her daily life. Inversely, some people with less severe fatigue feel they are enormously limited in their activities. Therefore it is important to search for the most appropriate tool for a particular dimension of fatigue.

In intervention studies evaluating strategies to reduce fatigue (medication, cooling, energy management etc.), one of the inclusion criteria is usually a certain degree of fatigue (impact). The most commonly used instruments for this purpose are the Fatigue Severity Scale or the (Modified) Fatigue Impact Scale. However, the administration of these scales takes some time (10–20 minutes) and they are often used as outcome measures as well.

Visual analogue scales (VAS) are frequently used to assess subjective feelings, like pain and fatigue. However, reports of research evaluating the scientific properties of the scales are sparse [4,5]. Besides, nearly every VAS is unique when different expressions are used.

We developed three potential visual analogue scales to rapidly assess the impact of fatigue in multiple sclerosis. This study evaluates the reproducibility and the validity of the scales and determines the most appropriate VAS for screening purposes.

## Methods

### Sample

The study ethics committee of the National MS Centre of Melsbroek approved the study, and informed consent was obtained from all participants.

Out of all in- and outpatients attending the rehabilitation service of the National MS Centre of Melsbroek (Belgium), an electronic randomization assigned 85 individuals with clinically definite MS to the study group. Subjects were included when adequate physical (upper limb) and cognitive functioning was demonstrated. The physical performance was determined by a minimum score of 30 or more on the self-care subscore of the Functional Independence Measure; adequate cognitive functioning was demonstrated by a Rao's neuro-psychological battery [6] of 3 or more. Sixty two individuals met all criteria and were included in the study. Twenty four employees of the centre volunteered as healthy controls, one of them did not perform the second assessment.

### Procedure

**Figure 1:**
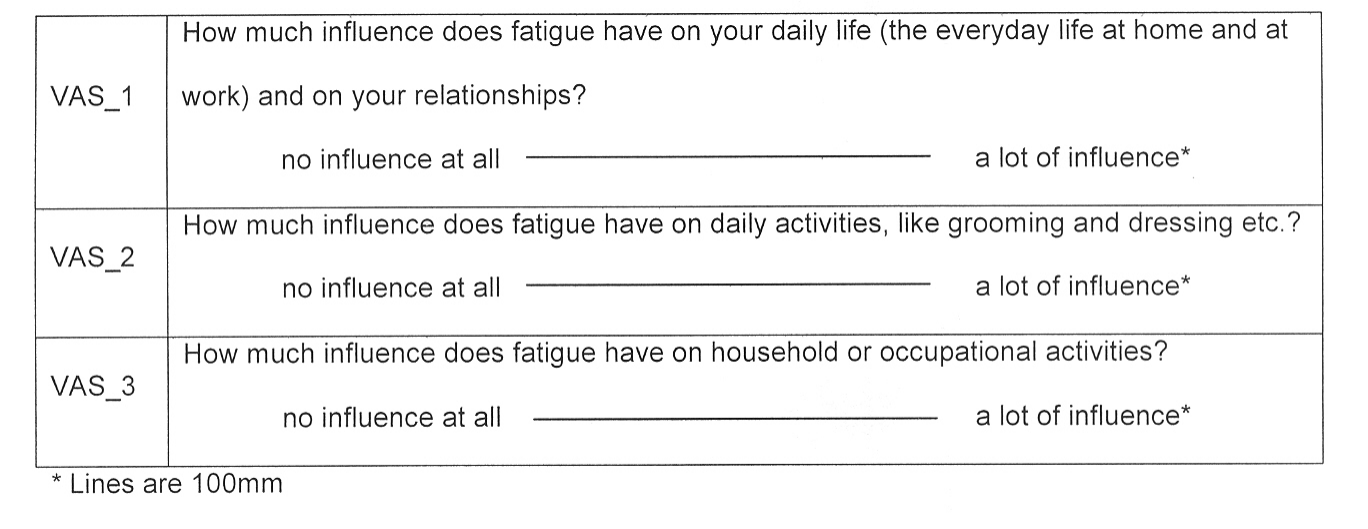
Visual Analogue Scales for the impact of fatigue.

Three Visual Analogue Scales to assess the impact of fatigue on daily life (VAS_1), on self care activities (VAS_2) and on household and occupation (VAS_3) were developed. The answer line of 100 mm ranges from "no influence at all" to "a lot of influence" (Figure 1).

The three VAS scales were administered simultaneously with the Modified Fatigue Impact Scale (MFIS), the Fatigue Severity Scale (FSS) and the fatigue subscale of the Guy's Neurological Disability Scale (GNDS), in a random order.

The Modified Fatigue Impact Scale assesses the impact of fatigue on daily functioning during the last four weeks. Subjects are requested to indicate the frequency of influence of fatigue in 21 situations (range 0–4), resulting in a total score and three subscores (physical, cognitive and psychosocial subscale) [7-9].

In the Fatigue Severity Scale (FSS), individuals have to rate their agreement (range 1–7) with nine statements concerning the severity, frequency and impact of fatigue on daily life [10].

The Guy's Neurological Disability Scale (GNDS) is a disability measure developed for use in multiple sclerosis; it consists of 12 categories, including fatigue. The fatigue score (range 0–5) is based on the presence and severity of fatigue and its impact during the last four weeks [11,12].

Divergent validity was considered with Kurtzke's Expanded Disability Status Scale [13], the Functional Independence Measure [14], the neuro-psychological battery of Rao [6,15] and Zung self-rating depression scale [16].

To evaluate the reproducibility of the scale, the measurement was repeated within three days in all subjects, at the same time of the day.

### Statistical analysis

All results were analyzed using the software package SPSS for Windows Standard Version 13, 2004. Differences between groups for variables at ordinal level were analyzed with a Mann-Whitney rank sum test. Differences in age were analyzed with t-test. All ordinal correlation analyses were performed with Kendall tau-b; otherwise a Pearson correlation coefficient was used. Reproducibility of the VAS scales was quantified with the intraclass correlation coefficient (ICC), based on one-way analysis of variance for repeated measurements [17]. The differences between mean scores of the two measurements with their 95% confidence interval were calculated.

A Receiver Operating Characteristic (ROC) curve analysis was used to determine the accuracy of the Visual Analogue Scales.

Results were considered statistically significant when p < .05.

## Results

### Sample characteristics

Demographic and clinical data of the sample are displayed in Table 1. Patient group and controls did not differ in age and gender distribution, but showed a significant difference in fatigue scores.

### Reliability

**Figure 2:**
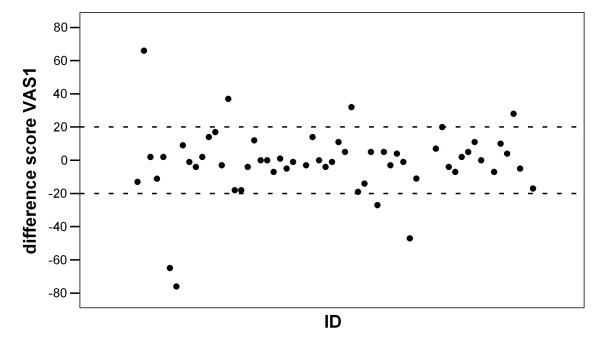
Difference in VAS_1 scores between first and second assessment.

**Figure 3:**
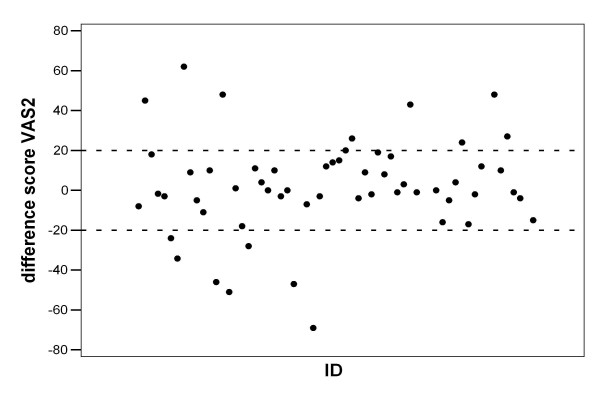
Difference in VAS_2 scores between first and second assessment.

**Figure 4:**
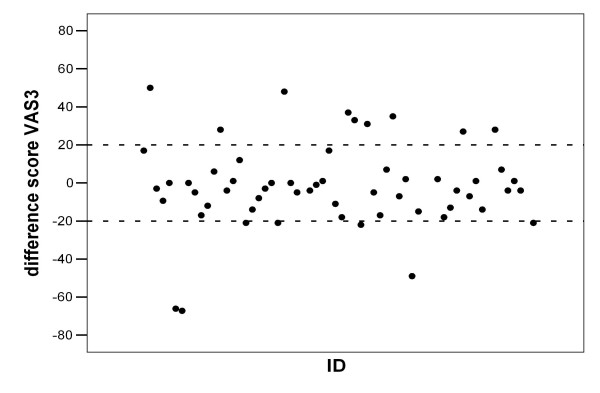
Difference in VAS_3 scores between first and second assessment.

Of the three visual analogue scales VAS_1 showed the smallest difference in raw scores in repeated assessment after three days (Figures 2, 3, 4). The intraclass correlation coefficient confirmed this observation (Table 2). Considering the total MS sample, the ICC was moderate for all scales, with values ranging between 0.68 and 0.69. In the subjects with better cognitive performance (RAO = 4), the ICC values were similar in all scales. In subjects with a RAO score of 3, the reproducibility of VAS_2 seemed reduced, whereas the other scales showed similar ICC's. Analysis of covariance did not reveal cognition as a covariate however. For all visual analogue scales no significant differences in raw scores between groups (RAO = 4 versus RAO = 3) existed.

### Validity

VAS_1 correlated low to moderately with all fatigue scales (values ranged from 0.28 to 0.48), whereas the correlation coefficients of VAS_2 and VAS_3 with the other scales did not reach higher levels than 0.37, indicating a weak association (Table 3). Considering the subscales of the Modified Fatigue Impact Scale (MFIS), mainly the physical subscale accounted for the association with the VAS scales. However, no significant relationships were found between VAS scores and physical performance, assessed with EDSS and the self-care domain of the Functional Independence Measure. Besides, neither of the VAS scores correlated with Zung depression scale or Rao's cognitive battery. Fifty-two percent of the subjects scored more than the mean depression score for depressed psychiatric outpatients (score 51) studied by Zung (1965) [16]. However, no significant differences in VAS scores existed between the non-depressed and depressed group.

### Accuracy

**Figure 5:**
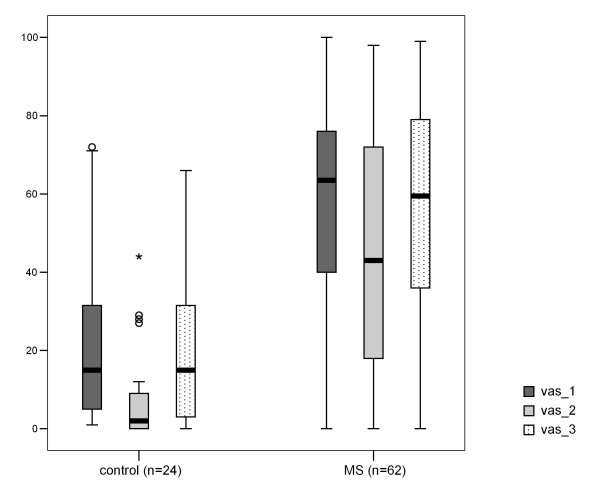
**Distribution of VAS scores in healthy controls and persons with MS**. Boxes represent 25–75% of the cases; whiskers are minimum and maximum values, lines: median values, °: outliers, *: extreme values. Differences between groups are statistically significant (Mann Whitney U test, p < 0.0001).

The distribution of VAS scores in healthy controls and persons with MS are displayed in Figure 5. All three scales were able to discriminate between both groups (p < 0.0001); VAS_2 was the strongest tool to classify healthy controls and subjects with MS (see also Additional file 1: Discrimination between healthy controls and subjects with MS: area under the ROC curve).

The subjects with a high level of fatigue were classified in the fatigued group, using the cut-off value 36 of the Fatigue Severity Scale (FSS) described in Flachenecker et al. (2002). The accuracy of the VAS to correctly classify subjects who are highly fatigued, was considered in an ROC analysis. This analysis revealed that VAS_1 was most appropriate for this purpose, with an area under the curve of .78, whereas this value was .68 for VAS_2 and .71 for VAS_3. A cut-off value on the VAS_1 of 59 displayed 76% sensitivity and 72% specificity to discriminate between fatigued and non-fatigued persons with MS (see Additional file 2: Discrimination between fatigued and non-fatigued subjects with MS: cut-off values). When using the critical value of the Modified Fatigue Impact Scale score to discriminate between persons with high impact of fatigue on their daily life (MFIS > 39) [18] and those with less influence of fatigue, the VAS_1 remained the strongest tool, with 81% sensitivity and 77% specificity at a cut-off value of 59 (see Additional file 3: Discrimination between high and low fatigue impact in subjects with MS: cut-off values).

**Figure 6:**
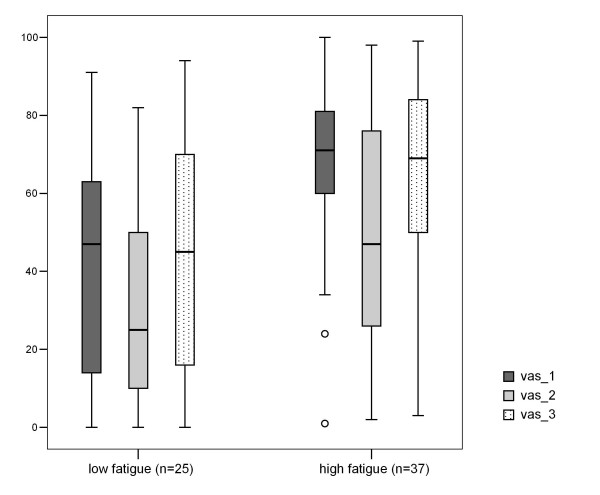
**Scores of the VAS scales in subjects with low versus high fatigue (based on FSS)**. Boxes represent 25–75% of the cases; whiskers are minimum and maximum values, lines: median values, °: outliers. Differences between groups are statistically significant (Mann Whitney U test, p < 0.0001).

**Figure 7:**
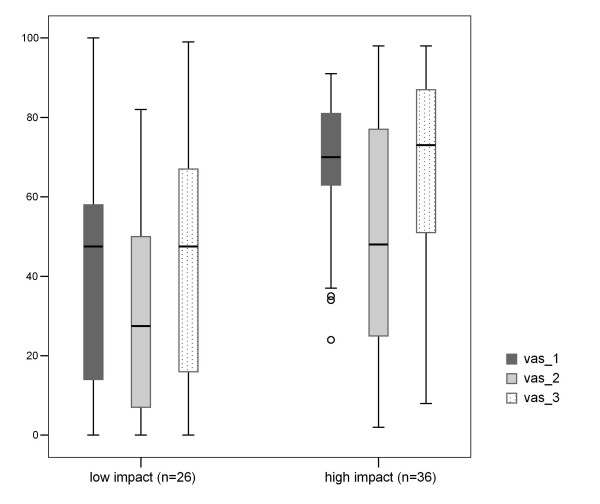
**Scores of the VAS scales in subjects with low versus high impact of fatigue (based on MFIS)**. Boxes represent 25–75% of the cases; whiskers are minimum and maximum values, lines: median values, °: outliers. Differences between groups are statistically significant (Mann Whitney U test, p < 0.0001).

Figures 6 and 7 show the distribution of the scores of all visual analogue scales. In contrast to VAS_2 and VAS_3, VAS_1 was able to discriminate between fatigue (impact) groups without substantial overlap of the interquartile range. However, based on rank scores, differences between groups were statistically significant in all three VAS scales (Mann Whitney U test, p < 0.0001).

## Discussion

This study aimed at finding a screening tool to assess rapidly the impact of fatigue on the daily life of subjects with multiple sclerosis. Three visual analogue scales have been developed and evaluated. The VAS that reflects all aspects of daily life (VAS_1) had the highest reproducibility and ability to detect people with high levels of fatigue (impact).

The reproducibility of all scales was moderate (0.68–0.69). Every millimeter of the scale can be considered as one unit of the score, therefore visual analogue scales are sensitive to a large variability. However, the difference in raw VAS_1 scores between the two points of measurement did not exceed 20% in most cases.

The correlations of VAS with other fatigue scales were low to moderate, which implies different dimensions of fatigue being assessed by various fatigue scales. Similar results were reported in other studies [3,8,18,19]. The weak correlation of the cognitive and psychosocial subscales of MFIS with all three VAS scales suggests that the visual analogue scales used in this study reflect the influence of fatigue on physical performance rather than on cognitive and psychosocial abilities. One possible explanation is that one does not always acknowledge the influence of fatigue on cognitive or psychosocial performance and therefore these aspects are not reflected in the VAS scores.

Of the three visual analogue scales, VAS_2 was most useful in discriminating between healthy controls and persons with MS. VAS_2 reflects the influence of fatigue on personal care, which is apparently lower in controls. Every human being experiences fatigue once in a while, but it becomes pathological when it interferes with daily life and certainly with self care. This idea is also generally stated in the definition of the MS Council that describes fatigue as the perception of "a subjective lack of physical and/or mental energy...to interfere with usual and desired activities" (p.2) [7].

Discrimination between high and low fatigue (impact) within the MS group was most accurate in VAS_1, particularly when MFIS was used to classify subjects. This is expected, of course, for the VAS_1 is a tool to assess the impact of fatigue, which can be different from the severity of fatigue quantified by the FSS. That is, individuals with a similar level of fatigue severity do not necessarily experience similar fatigue impact. Therefore both features should be assessed separately in order to plan intervention strategies. The VAS in this study is developed to detect persons with MS with high fatigue impact and, for example, can be used as inclusion criterion for research purposes. Especially when multiple data are collected (which may provoke fatigue), the proposed VAS has the advantage to provide a rapid impression of the level of fatigue impact. In clinical practice however, a more elaborate assessment of fatigue and the impact on daily life should be performed in order to provide a tailor-made intervention.

Although VAS_1 was the strongest scale to discriminate between fatigued and non-fatigued subjects, the difference with the other visual analogue scales was moderate. Additional research with these scales in other MS and control samples is needed to confirm our results.

A major concern in using self-report instruments is the applicability in people with cognitive disabilities. We selected individuals without major cognitive problems. When the subjects without cognitive problems and those with minor difficulties were analyzed separately, we found similar reproducibility rates of the visual analogue scales. Moreover, VAS did not correlate with cognitive performance in this sample.

Despite the high incidence of depression in our sample, VAS scores did not correlate with Zung's depression scale, whereas MFIS showed low correlation (0.3). Depression is often related to fatigue [20-27], but the causality of this relationship is not clear. In general, depressed individuals show higher fatigue levels; having disabling fatigue on its turn may culminate in depression. In our sample, no higher VAS-scores were found in the depressed group, however. These results support the construct validity of the VAS, i.e. assessing the impact of fatigue and not of depression.

A limitation of this study is the use of other self-report instruments (MFIS and FSS) to split the sample in a non-fatigued and a fatigued group. However, it is not straightforward to assess the symptom of fatigue otherwise. Several methods have been proposed to objectively measure fatigue, but these did not – or at best weakly – correlate with perception of the symptom ([28-32]. Fatigue probably consists of multiple dimensions (e.g. motor versus cognitive fatigue), that should be assessed by different techniques.

The sample in our study showed a large variability in EDSS scores (ranging from 3 to 8.5), and thus represented the physical performance of the MS population to a great extent. However, subjects in the lower boundaries of the EDSS (0–2.5) were not included due to site bias (only persons attending the rehabilitation centre entered the study). The results of this study can therefore not be generalized to the whole MS population.

## Conclusion

The Visual Analogue Scale for assessing the impact of fatigue on daily life (VAS_1) is a moderately reliable, though valid and useful tool to screen rapidly for fatigue impact in multiple sclerosis. A cut-off value of 59 satisfactorily classifies individuals having severe fatigue with a high impact on daily life. In clinical practice, a more comprehensive assessment of fatigue and the impact on daily life is recommended.

## Competing interests

The author(s) declare that they have no competing interests.

## Authors' contributions

DK conceived of the study, participated in the design, coordination and statistical analysis and drafted the manuscript. GN participated in the design and statistical analysis. MBD and MD participated in the design of the study and interpretation of data. EK participated in the design, coordination and statistical analysis. All authors critically appraised and approved the final manuscript.

## Pre-publication history

The pre-publication history for this paper can be accessed here:



## Supplementary Material

Additional file 1 Discrimination between healthy controls and subjects with MS. Receiver Operating Characteristic (ROC) curve analysis of the three VAS scales: area under the curve. Click here for file

Additional file 2 Discrimination between fatigued and non-fatigued subjects with MS. Receiver Operating Characteristic (ROC) curve analysis of the three VAS scales: cut-off values. Click here for file

Additional file 3 Discrimination between high and low fatigue impact in subjects with MS. Receiver Operating Characteristic (ROC) curve analysis of the three VAS scales: cut-off values. Click here for file

## Figures and Tables

**Table 1:** Demographic and clinical data of the sample

	Subjects with MS n = 62	Healthy controls n = 24	p value
Age (mean, SD)	52 ± 10.5	52 ± 14.0	0.59°
Gender (F/M)	29/33 (47/53%)	13/11 (54/46%)	0.54*
EDSS (Me, IQR)	6.5 (3–8.5)	-	
Type MS (PP/RR/SP/NA)	14/16/20/12	-	
VAS_1 (Me, IQR)	63 (39–76)	23 (5–60)	<0.0001*
VAS_2 (Me, IQR)	43 (18–73)	3 (0–20)	<0.0001*
VAS_3 (Me, IQR)	60 (35–79)	18 (3–51)	<0.0001*
Fatigue Severity Scale (Me, IQR)	48 (34–56)	23 (15–32)	<0.0001*
Modified Fatigue Impact Scale (Me, IQR)	43 (57–29)	24 (9–31)	<0.0001*
Guy's NDS, subscale fatigue (Me, IQR)	3 (1–3)	0 (0–3)	0.01*

PP: primary progressive, RR: relapsing remitting, SP: secondary progressive, NA: data not available

Me: median, IRQ: interquartile range

° t-test * Mann-Whitney U test

**Table 2:** Reproducibility of the VAS scales: intraclass correlation with 95% confidence interval

	**Total MS sample (n = 62)**	**Rao = 4 (n = 38)**	**Rao = 3 (n = 24)**
*VAS_1*	.69 (.53–.81)	.72 (.52–.85)	.67 (.31–.86)
*VAS_2*	.68 (.51–.80)	.74 (.54–.85)	.52 (.01–.79)
*VAS_3*	.69 (.53–.80)	.65 (.42–.80)	.74 (.42–.89)

Rao: Rao's cognitive battery

**Table 3:** Correlations with fatigue scales, Kendall's tau-b

	VAS_1	VAS_2	VAS_3
Fatigue Severity Scale	0,45**	0,27**	0,37**
Modified Fatigue Impact Scale (MFIS)	0,41**	0,23**	0,32**
MFIS_physical subscale	0,48**	0,29**	0,36**
MFIS_cognitive subscale	0,28**	0,17	0,23**
MFIS_psychosocial subscale	0,39**	0,19*	0,26**
Guy's Neurological Disability Scale, fatigue subscale	0,43**	0,27**	0,37**

* p < 0.05, ** p < 0.01

## References

[B1] Krupp LB (2003). Fatigue.

[B2] Pompeii LA, Moon SD, McCrory DC (2005). Measures of physical and cognitive function and work status among individuals with multiple sclerosis: a review of the literature. J Occup Rehabil.

[B3] Kos D, Kerckhofs E, Ketelaer P, Duportail M, Nagels G, D'Hooghe MB, Nuyens G (2003). Self-report assessment of fatigue in multiple sclerosis: a critical evaluation. Occupational Therapy in Health Care.

[B4] Guyatt GH, Townsend M, Berman LB, Keller JL (1987). A comparison of Likert and visual analogue scales for measuring change in function. J Chronic Dis.

[B5] Lee KA, Hicks G, Nino-Murcia G (1991). Validity and reliability of a scale to assess fatigue. Psychiatry Res.

[B6] Rao S (1990). A manual for the brief, repeatable battery of neuropsychological tests in multiple sclerosis.

[B7] Multiple Sclerosis Council for Clinical Practice Guidelines (1998). Fatigue and multiple sclerosis: evidence-based management strategies for fatigue in multiple sclerosis.

[B8] Kos D, Kerckhofs E, Nagels G, D'Hooghe BD, Duquet W, Duportail M, Ketelaer P (2003). Assessing fatigue in multiple sclerosis: Dutch modified fatigue impact scale. Acta Neurol Belg.

[B9] Kos D, Kerckhofs E, Carrea I, Verzo R, Ramos M, Jansa J (2005). Evaluation of the Modified Fatigue Impact Scale in four different European countries. Mult Scler.

[B10] Krupp LB, LaRocca NG, Muir-Nash J, Steinberg AD (1989). The fatigue severity scale. Application to patients with multiple sclerosis and systemic lupus erythematosus. Arch Neurol.

[B11] Sharrack B, Hughes RA (1999). The Guy's Neurological Disability Scale (GNDS): a new disability measure for multiple sclerosis. Mult Scler.

[B12] Nuyens G, Van Asch P, Kerckhofs E, Vleugels L, Duportail M, Ketelaer P (2002). The UK neurological disability scale: reliability and relation with the narcoms performance scale. Int J MS Care.

[B13] Kurtzke JF (1983). Rating neurologic impairment in multiple sclerosis: an expanded disability status scale (EDSS). Neurology.

[B14] Keith RA, Granger CV, Hamilton BB, Sherwin SS, Eisenberg MB and Grzesiak RC (1987). The functional independence measure: a new tool for rehabilitation. Advances in clinical rehabilitation, Vol 1.

[B15] Rao SM, Leo GJ, Bernardin L, Unverzagt F (1991). Cognitive dysfunction in multiple sclerosis. I. Frequency, patterns, and prediction. Neurology.

[B16] Zung WW (1965). A self-rating depression scale. Arch Gen Psychiatry.

[B17] Rankin G, Stokes M (1998). Reliability of assessment tools in rehabilitation: an illustration of appropriate statistical analyses. Clin Rehabil.

[B18] Flachenecker P, Kumpfel T, Kallmann B, Gottschalk M, Grauer O, Rieckmann P, Trenkwalder C, Toyka KV (2002). Fatigue in multiple sclerosis: a comparison of different rating scales and correlation to clinical parameters. Mult Scler.

[B19] Chipchase SY, Lincoln NB, Radford KA (2003). Measuring fatigue in people with multiple sclerosis. Disabil Rehabil.

[B20] Bakshi R, Shaikh ZA, Miletich RS, Czarnecki D, Dmochowski J, Henschel K, Janardhan V, Dubey N, Kinkel PR (2000). Fatigue in multiple sclerosis and its relationship to depression and neurologic disability. Mult Scler.

[B21] Lobentanz IS, Asenbaum S, Vass K, Sauter C, Klosch G, Kollegger H, Kristoferitsch W, Zeitlhofer J (2004). Factors influencing quality of life in multiple sclerosis patients: disability, depressive mood, fatigue and sleep quality. Acta Neurol Scand.

[B22] Kroencke DC, Lynch SG, Denney DR (2000). Fatigue in multiple sclerosis: relationship to depression, disability, and disease pattern. Mult Scler.

[B23] Randolph JJ, Arnett PA, Higginson CI, Voss WD (2000). Neurovegetative Symptoms in Multiple Sclerosis: Relationship to Depressed Mood, Fatigue, and Physical Disability. Arch Clin Neuropsychol.

[B24] Voss WD, Arnett PA, Higginson CI, Randolph JJ, Campos MD, Dyck DG (2002). Contributing factors to depressed mood in multiple sclerosis. Arch Clin Neuropsychol.

[B25] Chwastiak LA, Gibbons LE, Ehde DM, Sullivan M, Bowen JD, Bombardier CH, Kraft GH (2005). Fatigue and psychiatric illness in a large community sample of persons with multiple sclerosis. J Psychosom Res.

[B26] Pittion-Vouyovitch S, Debouverie M, Guillemin F, Vandenberghe N, Anxionnat R, Vespignani H (2006). Fatigue in multiple sclerosis is related to disability, depression and quality of life. J Neurol Sci.

[B27] Schreurs KMG, de Ridder DTD, Bensing JM (2002). Fatigue in multiple sclerosis - Reciprocal relationships with physical disabilities and depression. J Psychosom Res.

[B28] Krupp LB, Elkins LE (2000). Fatigue and declines in cognitive functioning in multiple sclerosis. Neurology.

[B29] Schwid SR, Thornton CA, Pandya S, Manzur KL, Sanjak M, Petrie MD, McDermott MP, Goodman AD (1999). Quantitative assessment of motor fatigue and strength in MS. Neurology.

[B30] Schwid SR, Tyler CM, Scheid EA, Weinstein A, Goodman AD, McDermott MP (2003). Cognitive fatigue during a test requiring sustained attention: a pilot study. Mult Scler.

[B31] Kos D, Kerckhofs E, Nagels G, Geentjens L (2004). Cognitive fatigue in multiple sclerosis: comment on Schwid SR, Tyler CM, Scheid EA, Weinstein A, Goodman AD and McDermott MR. Mult Scler.

[B32] Surakka J, Romberg A, Ruutiainen J, Virtanen A, Aunola S, Maentaka K (2004). Assessment of muscle strength and motor fatigue with a knee dynamometer in subjects with multiple sclerosis: a new fatigue index. Clin Rehabil.

